# Arthroscopic Marrow‐Stimulating Procedure for Full‐Thickness Knee Cartilage Lesions

**DOI:** 10.1002/atn2.70320

**Published:** 2026-11-03

**Authors:** Jyothiprasanth M., Jithin C. R., Rahul Subramaniam P. S., Akhil K. Thomas, Aghosh M. C.

**Affiliations:** ^1^ Department of Orthopedics Baby Memorial Hospital Kannur, Kerala India

## Abstract

Articular cartilage defects remain challenging to treat because cartilage is avascular, sparsely cellular and has limited intrinsic healing potential. If left untreated, these lesions may progress to degenerative osteoarthritis, resulting in persistent pain and functional limitation. Marrow stimulation techniques such as drilling and microfracture are commonly used to promote repair by accessing subchondral bone marrow elements. Although these procedures are simple and widely practiced, their clinical benefits are often limited to the short‐ to mid‐term, and they may disrupt the subchondral bone plate, potentially compromising future cartilage restoration options. Nanofracture represents an evolution of traditional microfracture, using smaller‐diameter (1 mm) and deeper (9 mm) perforations to enhance marrow access while minimizing trabecular bone compaction. This article describes an arthroscopic nanofracture technique using a curved guide and depth‐controlled wire, allowing precise, evenly spaced perforations and improved access to challenging regions such as the posterior femoral condyles and tibial plateau without added surgical morbidity. By preserving subchondral architecture and ensuring consistent marrow stimulation, nanofracture may improve the biological quality of repair tissue.

## Graphical abstract

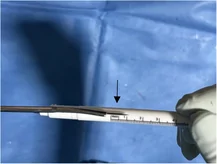

**VIDEO 1:** After standard arthroscopic preparation, portals are created for visualization and access without excess tissue trauma. A diagnostic arthroscopy through the Anterolateral portal assesses the cartilage damage. Nanofracturing instruments are then introduced. A curved guide commonly used for all‐suture anchor placement in shoulder labral repair at the 5‐o'clock position is inserted through the Anteromedial portal. Its curved design allows access to difficult regions such as the posterior femoral condyles and tibial plateau. A 1‐mm‐diameter guidewire with a depth guard is introduced. The depth guard ensures consistent penetration, set at 9 mm, the optimal depth for nanofracturing. Under arthroscopic visualization, the guidewire is advanced precisely into the defect. The surgeon creates evenly spaced microperforations in the subchondral bone to stimulate the release of bone marrow elements, including mesenchymal stem cells critical for cartilage regeneration. Video content can be viewed at https://doi.org/10.1002/atn2.70320.

Articular cartilage is a well‐structured tissue with complex biomechanical properties and remarkable durability. Cartilage defects pose significant challenges for orthopaedic surgeons and, if untreated, may progress to degenerative osteoarthritis, causing pain and loss of joint function.^1^ Reparative procedures like microfracture (MF) and other marrow stimulation techniques aim to penetrate the subchondral bone plate, filling defects with marrow elements to promote fibrocartilaginous repair, initially achieved through Kirschner wire drilling.^2^ Marrow stimulation, practiced for over 45 years through drilling, burring, and awls, enhances blood flow to the bone surface. While initially effective, these techniques often provide only short‐term relief and can damage the subchondral surface, with studies showing that MF is no more effective than debridement alone and may compromise future surgeries.

## SURGICAL TECHNIQUE

After standard arthroscopic preparation, portals are created for visualization and access without excess tissue trauma. A diagnostic arthroscopy through the Anterolateral portal assesses the cartilage damage. Nanofracturing instruments are then introduced (Figure 1). A curved guide commonly used for all‐suture anchor placement in shoulder labral repair at the 5‐o'clock position is inserted through the Anteromedial portal. Its curved design allows access to difficult regions such as the posterior femoral condyles and tibial plateau.

**FIGURE 1:**
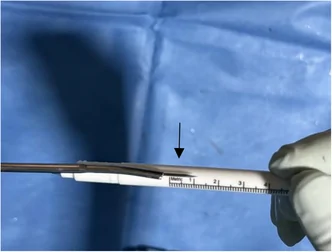
Curved guide (commonly used for all‐suture anchor placement in shoulder labral repair at the 5‐o'clock position) with custom‐made 1‐mm‐long K‐wire. The arrow showing the depth of the long K‐wire through the guide.

A 1‐mm‐diameter guidewire with a depth guard is introduced (Figure 2). The depth guard ensures consistent penetration—set at 9 mm, the optimal depth for nanofracturing. Under arthroscopic visualization, the guidewire is advanced precisely into the defect (Figure 3). The surgeon creates evenly spaced microperforations in the subchondral bone to stimulate the release of bone marrow elements, including mesenchymal stem cells critical for cartilage regeneration.

**FIGURE 2:**
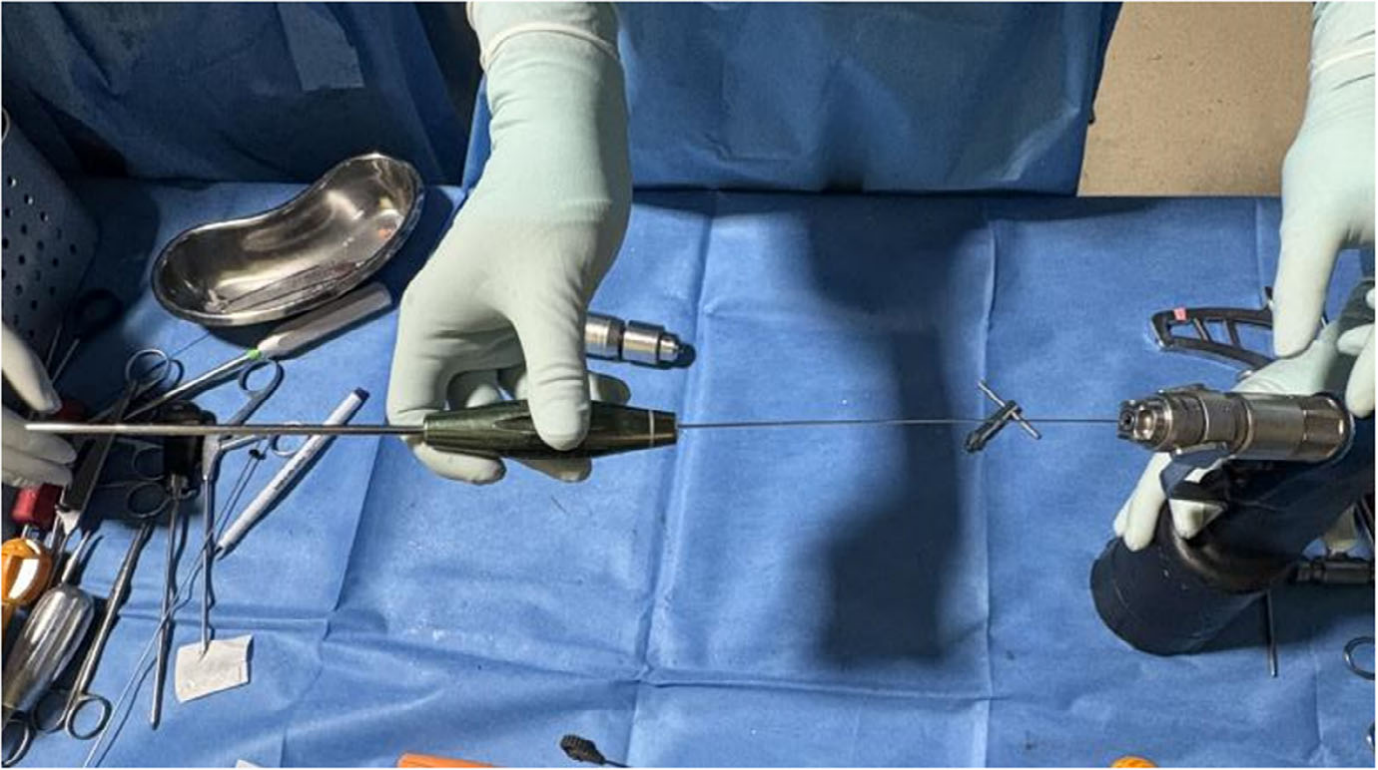
Custom‐made 1‐mm‐long K‐wire inserted through the curved guide with stopper to achieve 9 mm depth.

**FIGURE 3:**
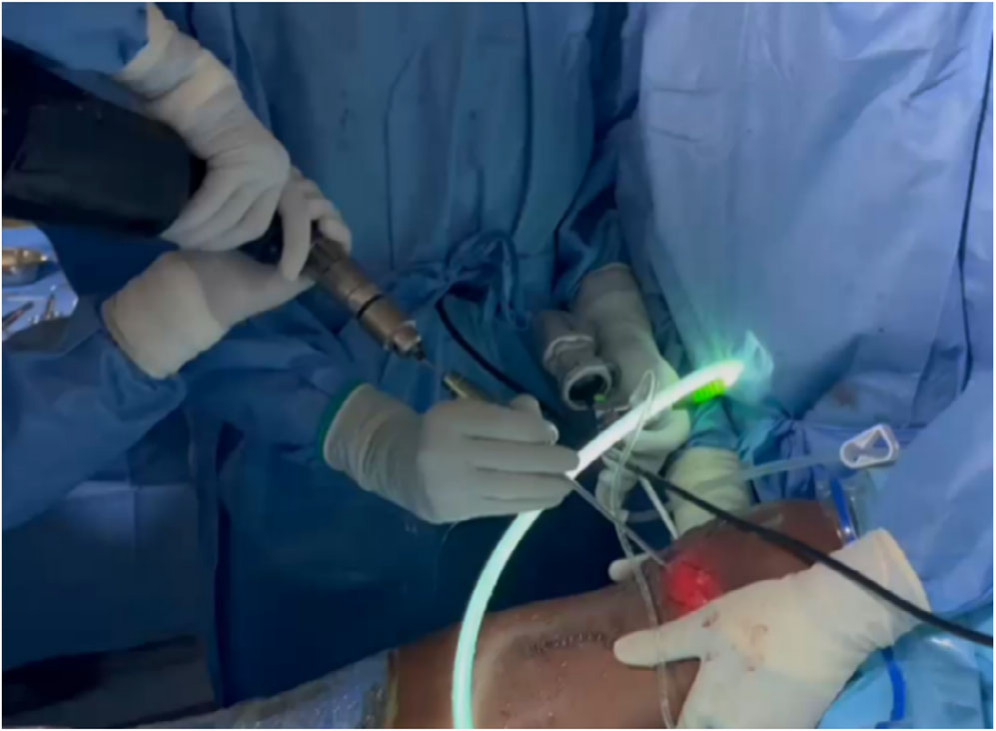
Insertion of curved guide and custom‐made 1‐mm‐long K‐wire with stopper through anteriomedial portal.

The curved guide improves access to posterior aspects of the femoral condyles and the tibial plateau (Figure 4), treating lesions that might otherwise require more invasive exposure. After completing the perforations, a final arthroscopic inspection is performed (Figure 5) to confirm an intact joint surface and the absence of complications (Video 1).

**FIGURE 4:**
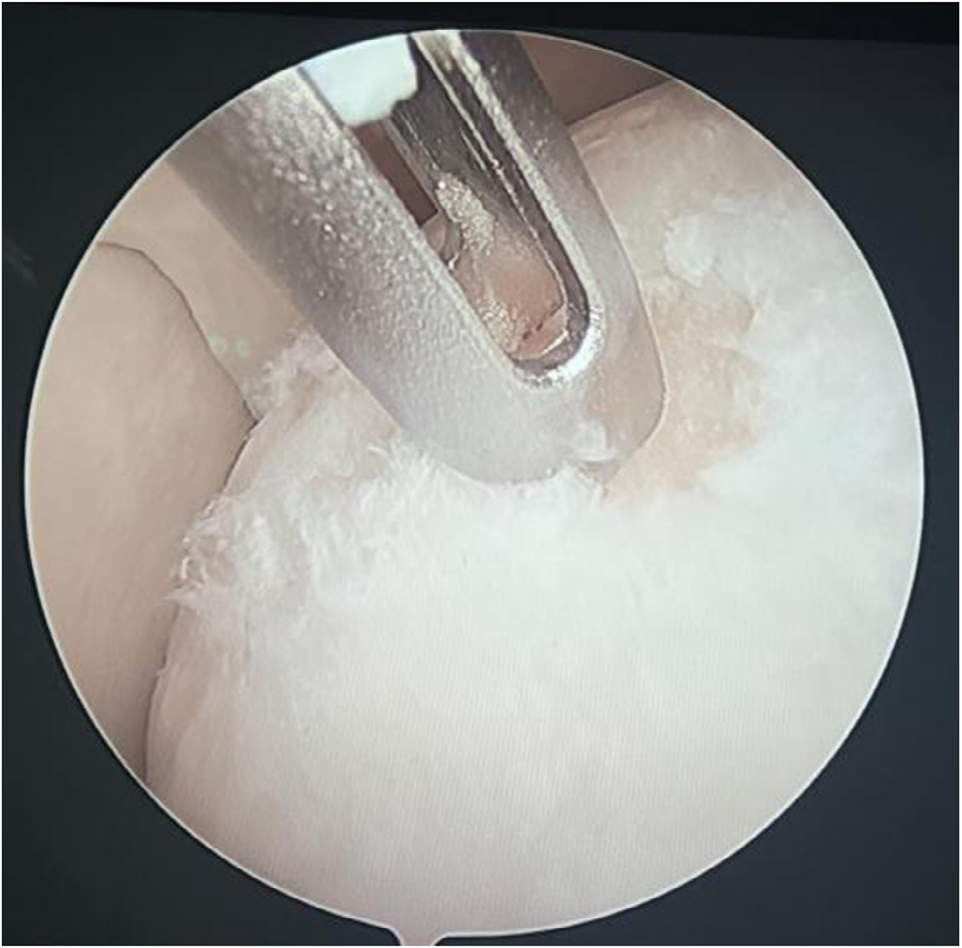
Arthroscopic image showing the cartilage defect in the femoral condyle with a curved guide and custom‐made 1‐mm‐long K‐wire with stopper placed over the defect.

**FIGURE 5:**
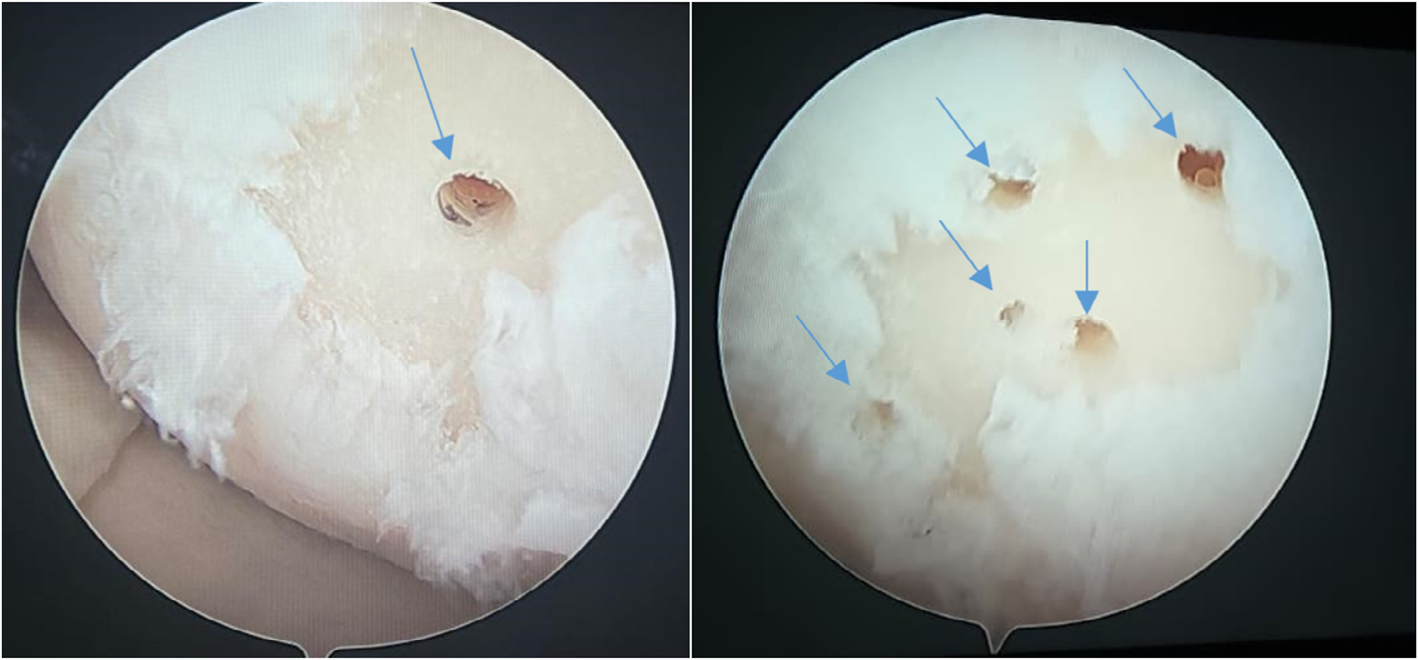
Drill hole of 1 mm diameter and 9 mm depth made at the posterior aspect of femoral condyle (arrow).

## DISCUSSION

Articular cartilage is a specialized avascular tissue providing a smooth, low‐friction surface for joint motion. It comprises 4 zones:

• Superficial (tangential) zone—tangential collagen fibers resist shear stress.
• Middle (transitional) zone—oblique fibers and proteoglycans transmit shear to compression.
• Deep (radial) zone—vertical fibers attach to the tidemark, distributing load.
• Calcified zone—includes the tidemark separating calcified from noncalcified cartilage.

As cartilage matures, its ability to regenerate diminishes because of the loss of multipotential cells and limited chondrocyte migration and proliferation. Mature articular cartilage has poor self‐repair capacity because of its avascularity, low cellularity, and limited matrix synthesis response.^3^

Cartilage injury treatment must address defect size, depth, and patient factors such as alignment, ligament stability, and rehabilitation.^4^ Nonsurgical management includes Nonsteroidal Anti‐inflammatory Drugs and physiotherapy for pain and function improvement. Severe degeneration or resistant cases may require surgical intervention such as the Osteochondral Autograft Transfer System, autologous chondrocyte implantation, or MF.^5^ Cell‐based therapies, particularly mesenchymal stromal cells, show promise for cartilage repair because mesenchymal stromal cells differentiate into chondrocytes and produce collagen and proteoglycans when combined with growth factors and scaffolds.^3^

Cartilage resurfacing procedures are broadly divided into marrow stimulation techniques and cell‐based techniques. Marrow stimulation methods like MF, abrasion chondroplasty, and subchondral drilling use marrow stem cells to form reparative fibrocartilage (mainly type I collagen). These are simple, cost‐effective, and provide short‐term symptom relief, although fibrocartilage is less durable and wear‐resistant than native hyaline cartilage.^4^

MF creates small subchondral bone perforations through arthroscopy to induce bleeding and clot formation, resulting in fibrocartilage repair tissue.^1^, ^4^ The technique stimulates bone marrow to attract mesenchymal stem cells that differentiate into fibrochondrocytes, forming a fibrocartilage clot.^6^ However, the repair tissue, primarily type I collagen, is biomechanically inferior to native type II collagen cartilage. Limited awl depth and trabecular compaction further reduce tissue quality.

Despite these drawbacks, MF remains popular because of its simplicity, low cost, and minimally invasive nature. Studies report short‐ to mid‐term improvement in pain and function, but long‐term durability is limited because of fibrocartilage degeneration, subchondral bone overgrowth, and declining outcomes over time.

To overcome these issues, the nanofracture technique was developed. It uses smaller‐diameter and deeper perforations (1 mm × 9 mm), causing less subchondral bone compaction and better preservation of trabecular structure compared with MF. The penetrator's design minimizes surface disruption while reaching a consistent depth 3 times deeper than traditional methods, improving marrow stimulation and cellular recruitment.

The curved guide used in nanofracture offers a major advantage by accessing hard‐to‐reach lesions such as the posterior femoral condyles and the tibial plateau without requiring extensive surgical exposure. The depth guard ensures uniform penetration, providing reliable stimulation of bone marrow elements like mesenchymal stem cells. Evenly spaced microperforations promote uniform cartilage healing and enhance the quality of the repair tissue.

The procedure's minimally invasive nature results in less tissue trauma, better subchondral bone preservation, and precise depth control, translating into improved outcomes. A final arthroscopic check confirms adequate perforation and the absence of complications. There are advantages and disadvantages when compared with MF (Tables 1 and 2).

**TABLE 1:** Advantages and Disadvantages

**Advantages**	**Disadvantages**
Minimally invasive arthroscopic procedure	Repair tissue is fibrocartilage (biomechanically inferior to hyaline cartilage)
Widely accessible technique	Outcomes dependent on surgical precision and lesion selection
Uses small‐bore drill → reduced subchondral bone compaction	Does not restore native cartilage architecture
Controlled depth penetration improves marrow access	Risk of incomplete or uneven marrow stimulation
Preserves trabecular architecture better than traditional microfracture	Limited evidence on long‐term durability
Evenly spaced perforations promote uniform healing response	May not be suitable for large or advanced degenerative lesions
Allows access to difficult areas (posterior femoral condyles, tibial plateau)	
No requirement for expensive implants or cell‐based therapies	

**TABLE 2:** Pearls and Pitfalls

**Pearls**	**Pitfalls**
Ensure thorough debridement of unstable cartilage before marrow stimulation	Inadequate debridement leads to poor integration of repair tissue
Use a small‐bore drill with controlled depth (≈8‐10 mm) for optimal marrow access	Shallow perforations → inadequate marrow stimulation
Maintain perpendicular orientation of awl to subchondral bone	Oblique penetration may cause trabecular damage or ineffective channels
Create evenly spaced perforations (3‐4 mm apart)	Overcrowding perforations weakens subchondral plate
Use curved guides for access to posterior or difficult‐to‐reach lesions	Poor access may result in incomplete treatment of lesion
Confirm “marrow fat droplets/bleeding” as endpoint	Failure to confirm marrow access reduces biological response
Proper patient selection (small, focal full‐thickness lesions)	Poor outcomes in diffuse osteoarthritis or malalignment

Compared with MF, nanofracture provides deeper marrow access, consistent perforation, reduced bone damage, and better trabecular preservation. These features enhance the biological repair potential and may improve long‐term clinical results.

## DISCLOSURES

The authors (J.M., J.C.R., R.S.P.S., A.K.T., A.M.C.) declare that they have no known competing financial interests or personal relationships that could have appeared to influence the work reported in this article.
